# Symbiosis extended: exchange of photosynthetic O_2_ and fungal-respired CO_2_ mutually power metabolism of lichen symbionts

**DOI:** 10.1007/s11120-019-00702-0

**Published:** 2019-12-31

**Authors:** Marie-Claire ten Veldhuis, Gennady Ananyev, G. Charles Dismukes

**Affiliations:** 1 Water Resources Section, Delft University of Technology, Stevinweg 1, 2628CN Delft, The Netherlands; 2 Waksman Institute of Microbiology, Rutgers University, 190 Frelinghuysen Rd, Piscataway, NJ 08854 USA; 3 Department of Chemistry and Chemical Biology, Rutgers University, 610 Taylor Rd, Piscataway, NJ 08854 USA

**Keywords:** Algae, Fungi, Lichens, Metabolism, Oxygenic photosynthesis, Respiration, Symbiosis

## Abstract

Lichens are a symbiosis between a fungus and one or more photosynthetic microorganisms that enables the symbionts to thrive in places and conditions they could not compete independently. Exchanges of water and sugars between the symbionts are the established mechanisms that support lichen symbiosis. Herein, we present a new linkage between algal photosynthesis and fungal respiration in lichen *Flavoparmelia caperata* that extends the physiological nature of symbiotic co-dependent metabolisms, mutually boosting energy conversion rates in both symbionts. Measurements of electron transport by oximetry show that photosynthetic O_2_ is consumed internally by fungal respiration. At low light intensity, very low levels of O_2_ are released, while photosynthetic electron transport from water oxidation is normal as shown by intrinsic chlorophyll variable fluorescence yield (period-4 oscillations in flash-induced Fv/Fm). The rate of algal O_2_ production increases following consecutive series of illumination periods, at low and with limited saturation at high light intensities, in contrast to light saturation in free-living algae. We attribute this effect to arise from the availability of more CO_2_ produced by fungal respiration of photosynthetically generated sugars. We conclude that the lichen symbionts are metabolically coupled by energy conversion through exchange of terminal electron donors and acceptors used in both photosynthesis and fungal respiration. Algal sugars and O_2_ are consumed by the fungal symbiont, while fungal delivered CO_2_ is consumed by the alga.

**Electronic supplementary material:**

The online version of this article (10.1007/s11120-019-00702-0) contains supplementary material, which is available to authorized users.

## Introduction

Symbiotic relations have been vital throughout evolution to create new forms of life and support survival in challenging environments (Margulis and Fester 1991). Yet, knowledge of the physiological co-dependencies that define symbiotic relationships remains superficial in many respects. Lichens are a symbiosis of a fungus (mycobiont) and at least one green alga or cyanobacterium (photobiont) (Nash 2008). They are famous for their ability to tolerate desiccation, which enables them to survive in water-stressed environments (Kranner et al. 2008). Unlike plants, lichens lack vascular organs to directly control their water loss or uptake, which is termed poikilohydry (Proctor and Tuba 2002). Their water content equilibrates with atmospheric conditions and as a result, lichens range between desiccated and water-saturated states on a daily basis throughout much of their lifetime. This implies that their photosynthetic activity, respiration and net biomass growth is restricted to brief periods of time, in response to water uptake during intermittent periods of rainfall, dew formation (Palmqvist 2000; Lidén et al. 2010) or, for species containing algal photobionts, high relative humidity levels (Lange et al. 1986). The photobiont is suggested to gain higher tolerance to desiccation from the symbiosis, preventing denaturation of many biopolymers and organelles. Multiple publications have documented that the photobiont within intact lichen bodies exhibit higher tolerance to desiccation stress compared to freshly isolated photobionts (O’Hara et al. 1983; Sass et al. 1995; Kosugi et al. 2009). The mycobiont receives excess sugars produced by the photobiont and excreted into the fungal filaments (Eisenreich et al. 2011). The elevated content of sugars serves as food to the fungal host and as the key osmolytes that protect both the algal and fungal tissues from loss of structural water from biomolecules during desiccation (Eisenreich et al. 2011; Green et al. 2011). In addition to water and sugars, the availability of O_2_ and CO_2_ gases is vital to support respiration and photosynthesis, respectively, for lichen symbiosis. However, the sources of O_2_ and CO_2_ gases and the mechanisms controlling their internal delivery and exchange have not been widely investigated and, until now, have been assumed to originate exclusively from environmental sources.

### Lichen photosynthetic activity in relation to gas transport and hydration state

Photosynthetic activity in lichens is coupled to their hydration state, the variability of which in turn strongly depends on climatic conditions. Lange et al. (1993) distinguish four types of photosynthetic response to water content based on a study of 22 lichen species from a temperate rainforest in New Zealand. Photosynthetic response, based on measurements of CO_2_ exchange rates, to high water content varied from no depression, a little depression, to large depression and even negative CO_2_ exchange at high water content, while a fourth type showed an optimum net photosynthesis at medium water content with low activity at both low and high water contents. A wide range of water contents was found in the field samples, with species varying from 357 to 3360% maximal water content (as  % of dry weight) and 86 to 1300% water content for optimal photosynthesis (Lange et al. 1993). The decrease in CO_2_ exchange rates in some species has been interpreted as arising from growing thallus diffusion resistance to atmospheric CO_2_ under supersaturation conditions (Coxson et al. 1983; Lange et al. 1993; Máguas et al. 1995; Lange and Green 1996). Early studies found differential CO_2_ exchange from the upper and lower cortex with the atmosphere, suggesting an important role of the medulla in gas transport (Green et al. 1981). Microscopy studies have revealed hydrophobic layers covering fungal filaments within the medulla and extending over algal cells. These layers overlay a thicker hydrophilic polyglucan layer postulated as water transport zone, while the outer hydrophobic layers were postulated to help maintain gas-filled inter-filament spaces in the thallus interior for gas transport (Honegger and Haisch 2001; Honegger 2012). These studies have highlighted some of the conditions affecting photosynthetic response in relation to gas transport in lichens, yet to the authors’ knowledge, no previous studies have examined the possibility of CO_2_ and O_2_ exchange between lichen symbionts.

In this study, we investigate whether exchange of O_2_ and CO_2_ produced by algal photosynthesis and fungal respiration, respectively, plays a role in the lichen symbiosis. We investigate lichen *Flavoparmelia caperata*, a medium-to-large foliose lichen with rounded lobes, measuring 3–8 mm wide, growing on bark of deciduous trees, colored distinctly gray when dry and green when wet. We used time-resolved oximetry to monitor light-induced O_2_ evolution (extracellular), which represents the flux of photosynthetic electron transport from water oxidation at the Photosystem II-Water Oxidation Complex (PSII-WOC). We also monitor PSII charge separation and water oxidation yield using intrinsic chlorophyll variable fluorescence yield (Fv/Fm) which is independent of possible O_2_ consumption. We aim to answer the following questions: How do algal O_2_ production and dark respiration rates change upon exposure to multiple light intensities, in aerobic and anaerobic conditions? How do internally produced O_2_, sugars and CO_2_ influence subsequent O_2_ production and consumption rates in response to prior illumination and dark periods?

This study provides the first evidence for functional O_2_ and CO_2_ exchange between algal and fungal tissues. This discovery extends the earlier understanding of lichen symbiosis beyond exchange of photosynthetic sugars and water to encompass the gaseous products that literally power both types of energy production through fungal respiration (via algal O_2_) and algal photosynthesis (through fungal CO_2_).

## Materials and methods

### Preparation of lichen samples

*Flavoparmelia caperata*, a symbiosis between an ascomycete fungus and the green algal photobiont *Trebouxia gelatinosa* (Ahmadjian 1993), was collected from the bark of mature maple trees, approximately 1 to 3 m above the soil in Princeton, New Jersey, USA. The region has a humid subtropical climate, average annual precipitation ranging from 1100 to 1300 mm, uniformly spread through the year. Thallus samples were stored for no more than 1 week under low light conditions at room temperature and 40–50% relative humidity. Disk-shaped samples of 4 mm diameter with thickness of approximately 70 μm were cut from a terminal lobe of a lichen thallus, the youngest portion of the lichen thallus, where the level of photochemical activity is typically higher than in the central part and where no dark lower cortex has yet been formed (Baruffo et al. 2008). Lichen samples were immersed in water for about 30 min and inserted into a water-filled cuvette (Clark electrode) or shaken dry and mounted into the cuvette (customized Clark-type rate electrode that consumes O_2_). This protocol achieves water-saturation as reported by Lange et al. (1993), Lange and Green (1996).

### Oxygen production and respiration from lichen under aerobic and anaerobic conditions

Two different oximetry methods were used to measure O_2_ concentration. A commercial Clark-type sensor comprised of a Teflon-covered Pt electrode (Hansatech, model DW-1/AD) was used to measure O_2_ concentration released from samples immersed in a microcell (1 ml volume, 4 mm diameter). The Clark electrode has a thick membrane (~ 10 µm) and slow response that directly measures O_2_ concentration without significant consumption from the sample chamber over time. Light-induced O_2_ signals were produced using a LED light source (5 W, 655 nm, at 800 μmol m^−2^ s^−1^ light intensity).

Lichen disks, once inserted into the cuvette of the Clark electrode, were sealed from air and stirred by magnetic bar at 500 rpm. The O_2_ concentration in the cuvette was recorded immediately, starting at aerobic conditions (O_2_ saturation in water, ~ 255 μM). Then lichen samples were alternatingly exposed to dark pre-conditions, subsequent light and subsequent dark conditions until all O_2_ was removed. After 100-min dark exposure to consume all O_2_ from the chamber, the same experiment was repeated at near-anaerobic initial conditions. Oxygen concentration was continuously measured as lichen disks were alternatingly exposed to dark and light conditions, the O_2_ data acquisition rate was 10 per second.

### Transient oxygen flux from lichen under high and low light intensities

A custom-built rate electrode comprised of a Pt–Ir alloy was used for measurement of O_2_ flux released from samples immersed in a thin-layer microcell (10 µl volume, 4 mm diameter) (Ananyev et al. 2016a, b). An ultra-thin membrane (~ 1 µm) was used that responds about 5 × times faster to changes in O_2_ concentration compared to the commercially available Clark electrodes, enabling measurement of faster kinetic processes, at sub-seconds scale (~ 0.1–0.3 s). This technique enables observation of O_2_ transients coupled to electron acceptors within PSII, PSI, and CO_2_ assimilation in the Calvin cycle. This behaves as a rate electrode which consumes O_2_ from the sample chamber and when the chamber is sealed, the small volume and large area allows anaerobic conditions to be established rapidly. The units for this electrode are in nA (current), which is directly proportional to the amount of O_2_ consumed per unit time by the electrode. The measured current is produced by O_2_ that is not consumed by the sample in the sealed chamber.

Using this electrode, O_2_ flux was measured from an intact lichen disk (4 mm diameter), exposed to continuous illumination from a red LED light source (655 nm) at two different light intensities of 70 and 800 μmol m^−2^ s^−1^ and in two different orientations (upper and bottom surface exposed to the O_2_ electrode). Each continuous illumination period lasted 90 s, after which the light source was turned off, while measurements continued for another 150 s. The initial (1st) illumination period was preceded by 30-min dark adaptation, after which 10 to 50 illumination periods were applied, separated by 10-min dark time between each illumination. The O_2_ data acquisition rate was 10 per second.

Using the same custom-built rate electrode, the O_2_ yield was measured from individual light-saturating flashes (STFs), each 50 microseconds in duration and delivered at a frequency of 0.5 Hz in a train of flashes produced by the same LED (*λ* = 655 nm). The O_2_ current was integrated between flashes to obtain the yield. This classic method enables observation of period-4 oscillations in O_2_ yield known to be produced by all oxygenic phototrophs, including free-living algae (Ananyev et al. 2016a, b). O_2_ flash yields in response to STFs were measured from lichen samples and separately from the isolated algal cells, after removal of the cells from the lichen sample by scrapping the lichen surface and re-suspending in BG11 medium, followed by 10-min gravitation precipitation to separate fungal from algal cells.

### Chlorophyll variable fluorescence yield

Induction of chlorophyll variable fluorescence yield was performed with a homebuilt Fast Repetition Rate (FRR) fluorometer utilizing a laser diode excitation source (*λ*_max_ = 655 nm) at a maximal flash intensity of 32,000 µmol m^−2^ s^−1^ (Ananyev and Dismukes 2005). It generates a series of 1-µs “flashlets” separated by approximately 1 µs. Approximately 25 flashlets comprise a single-turnover flash (STF). Each STF is capable of saturating the charge separation quantum yield of PSII in > 95% of the reaction centers. This occurs when the primary electron acceptor, plastoquinone-A (Q_A_), is reduced to the semiquinone Q_A_^−^. This closes the reaction center to further charge separation and increases the fluorescence emission yield from its dark-adapted minimum (Fo), arising from antenna chlorophyll emission, to its maximum emission (Fm), arising from both antenna and reaction center emission. The ratio Fv/Fm = (Fm − Fo)/Fm is directly proportional to the quantum yield of primary charge separation in PSII reaction centers (Kolber et al. 1998). When a train of STFs are applied to a dark-adapted sample, the transient Fv/Fm amplitude oscillates with period-4 cycle of flashes. The amplitude of these oscillations and their dependence of the flash rate provide a quantitative measure of the water oxidation activity of PSII without measuring O_2_ yield (Ananyev and Dismukes 2005). The transient amplitude dampens to a steady-state, light-adapted level which reflects the photochemical efficiency of PSII turnover. Note that Fv/Fm values measured by the FRR technique are typically lower than those measured using a PAM fluorometer, as they are associated with photoreduction of Q_A_^−^ by an STF, while the PAM signal is registered after reducing both Q_A_^−^ and the entire PQ pool (as PQH_2_). The FRR method of Chl fluorescence induction has been extensively applied in numerous studies of PSII in algae and in lichens (Kolber et al. 1998; Ananyev and Dismukes 2005; Fadeev et al. 2012; Ananyev et al. 2016a, b; Vinyard et al. 2018).

## Results

### Oxygen production and respiration rates under initial aerobic and anaerobic conditions

**Fig. 1:**
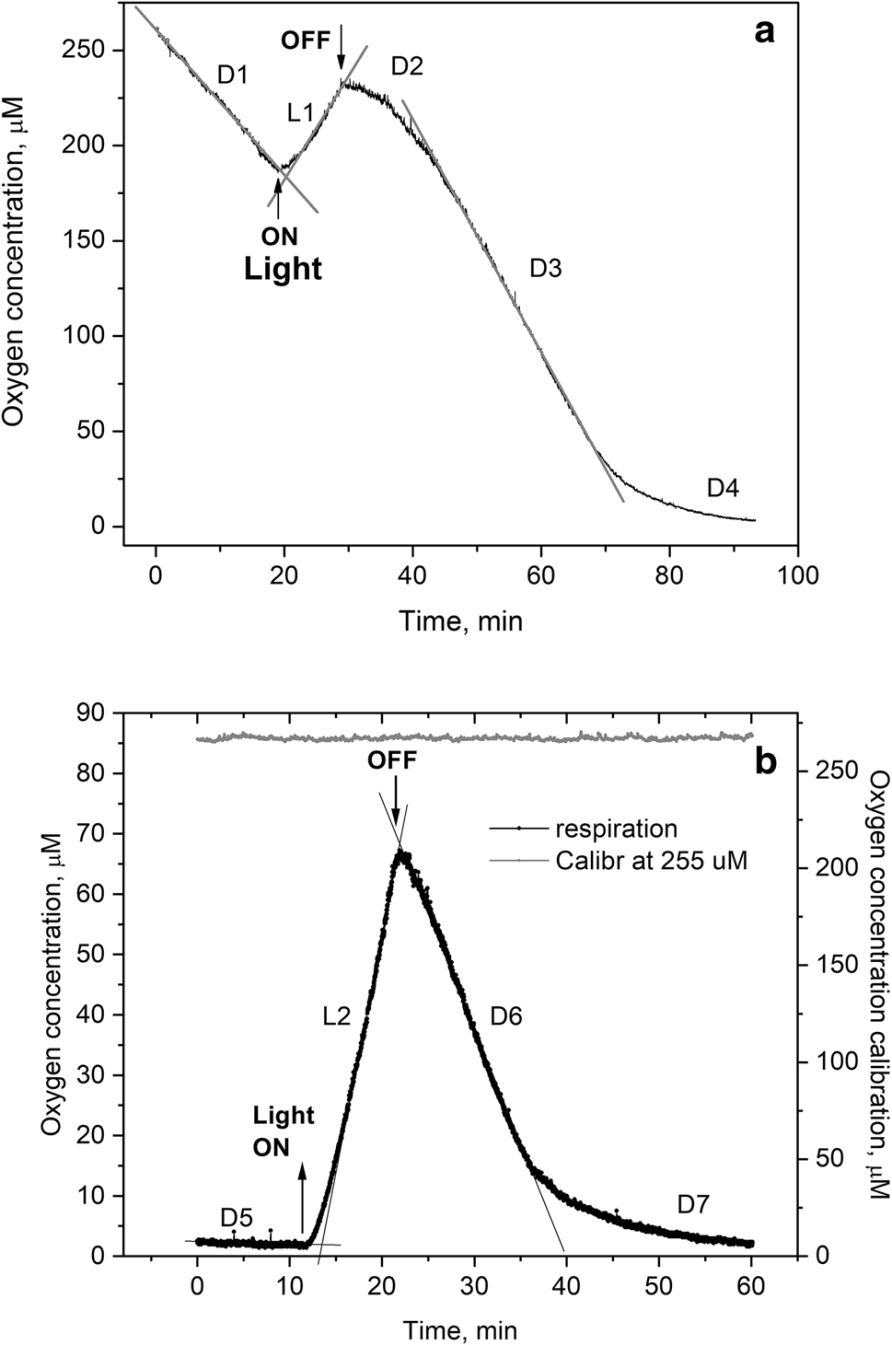
Evolution of extracellular O_2_ concentration in Clark cell chamber (1 ml) holding 13 lichen disks (4 mm diameter each) and stirred vigorously (500 rpm). L1, L2, D1 to D7 indicate O_2_ production resp. consumption rates under light and dark conditions. **a** Initial conditions aerobic, at O_2_ saturation in water (O_2_ ~ 255 μM). At time zero begins 20-min dark, followed by 10-min illumination at light intensity 800 μmol m^−2^ s^−1^ followed by 65-min dark (total time 95 min). **b** Initial conditions anaerobic, produced by 100-min pre-measurement dark exposure. At time zero begins 12-min dark time, followed by illumination for 10 min, followed by 38-min dark (total time 60 min). Second trace (2nd *y*-axis) shows electrode air-saturated water calibration over 60 min at 255 μM

Under initial air-saturated aerobic conditions in the dark, the O_2_ concentration decreases linearly over time, corresponding to a respiration rate of approximately − 215 μM h^−1^ for 13 lichen sample disks immersed in air-saturated water (Fig. 1a, trace D1). Net O_2_ production upon light exposure is approximately 275 μM h^−1^ (Fig. 1a, trace L1). Respiration in the dark, after illumination, increases to − 340 μM h^−1^ (Fig. 1a, trace D3). As light is turned off and O_2_ production ceases, respiration rate responds in three stages, as samples readapt to dark conditions. First, a slow net increase in respiration rate occurs for about 7–8 min (Fig. 1a, trace D2), followed by a period of constant respiration rate (− 340 μM h^−1^, Fig. 1a, trace D3), almost 60% higher compared to the initial, fully aerobic, dark-adapted conditions (− 215 μM h^−1^). The gross O_2_ production rate (μM h^−1^) is 490 before and 615 after light exposure, calculated as the difference between net O_2_ production and respiration rates before and after light exposure, respectively, assuming mitochondrial respiration rates continue at the same rate in light as in darkness before or after illumination. The linearity of regions D1 and D3 indicates that the respiration rates are independent of the varying O_2_ concentrations over these ranges. Finally, the respiration rate decreases exponentially below ~ 25 μM O_2_ concentration (Fig. 1a, trace D4), the threshold below which O_2_ availability limits the respiration rate.

Repeating the same experiment on the sample after attaining full anaerobic conditions (after 100-min dark exposure, consuming all intra- and extracellular O_2_) enables determination of net O_2_ production and respiration rates, independent of externally available O_2_. The net O_2_ production rate is approximately constant for 10 min at 375 μM h^−1^, at which point the light was turned off (Fig. 1b, trace L2). Higher net O_2_ production rate compared to the initial dark-adapted sample at full aerobic conditions (375 versus 275 μM h^−1^) can be explained, in principle, by lower fungal respiration and/or higher gross O_2_ production rates. The respiration rate in the dark (D6) after illumination (L2), is − 240 μM h^−1^, about 11% higher compared to that under initial aerobic conditions (− 240 versus − 215 μM h^−1^), yet considerably lower than that under aerobic conditions after light exposure (− 240 versus − 340 μM h^−1^). The gross O_2_ production rate is approximately 615 μM h^−1^ (L2–D6), essentially identical to that under initial aerobic conditions (L1–D3). The gross photosynthetic O_2_ production rate equates to 20 μM g^−1^ dwt s^−1^ when normalized to dry weight. The kinetic response during transitions from dark-to-light and light-to-dark differs dramatically for aerobic versus anaerobic samples, being much sharper for the initially anaerobic sample. The respiration rate decreases exponentially below ~ 15 µM O_2_ (Fig. 1b, trace D7).

Table 1 summarizes O_2_ production and respiration rates for the sample shown in Fig. 1 and three other thallus disk samples, taken from different lichen leaves (experimental data provided in Supporting Material, SI.1). Observed net O_2_ production rates are consistently higher under initial anaerobic, following dark respiration that consumes all O_2_, compared to initial aerobic conditions (L2 versus L1). Respiration rates are typically higher after illumination than in aerobically dark-adapted samples (D3 versus D1, clear rate increase in samples 1, 2, and 4; similar rates in sample 3). Gross O_2_ production rates (L1–D3 and L2–D6) vary as a result of biological variability of field samples as expected, however, they are very similar for initial aerobic versus anaerobic conditions (difference 0–13%). Results across biological samples illustrate that respiration rates increase following illumination and that net photosynthetic O_2_ production rates increase following respiration, while preserving an approximately constant gross production rate. This indicates that the two processes are metabolically linked and are capped at peak rates at high light intensity (800 μmol m^−2^ s^−1^).

**Table 1:** Net O_2_ production and dark consumption (respiration) rates (μM h^−1^) for sample from Fig. 1 and three other lichen samples

Initial conditions	Aerobic (μM h^−1^)				Anaerobic (μM h^−1^)		
O_2_ response trace	D1	L1	D3	L1–D3	L2	D6	L2–D6
Sample nr. (1)	− 215	275	− 340	615	375	− 240	615
nr. (2)	− 660	550	− 1090	1640	–	–	–
nr. (3)	− 460	390	− 455	845	518	− 295	813
nr. (4)	− 385	320	− 580	900	392	− 650	1042

D1 and D3: respiration rate in dark, aerobic conditions, before and after illumination, L1 and L2: O_2_ production rate during illumination, in aerobic and anaerobic conditions, respectively. D6: respiration rate after illumination L2, in initially anaerobic conditions. L1–D3, L2–D6: Gross O_2_ production rates (light–dark)

### Transient oxygen flux at different light intensities and sample orientation

**Fig. 2:**
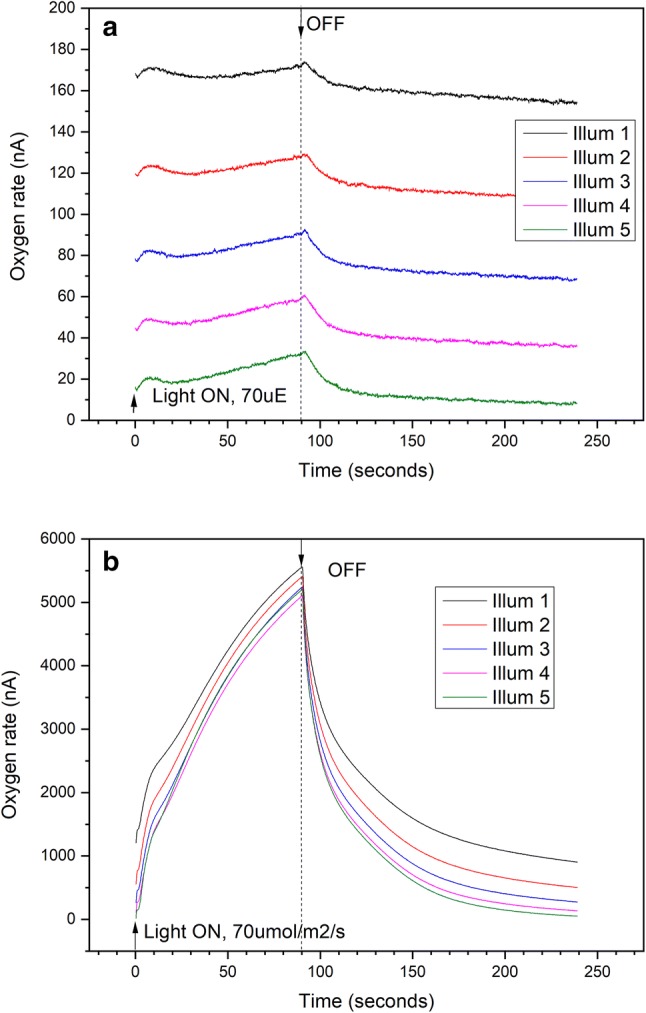
Time-resolved oxygen measured by rate electrode (custom Clark electrode). Each continuous illumination period lasts 90 s, separated by 10-min dark time between each illumination. Light intensity 70 μmol m^−2^ s^−1^. **a** Lichen disk, 4 mm diameter, top of lichen disk facing electrode, 1 μl BG11 medium added for hydration of sample and electrode. **b** Green algal Chlorella vulgaris immersed in BG11 medium, 5 μl sample volume

The effect of illumination on O_2_ fluxes was further investigated using the custom O_2_ rate electrode. This enables identification of transient changes in oxygen flux, the electrode responding rapidly to changes in O_2_ in the chamber because of its thinner membrane and tiny volume. Figure 2 shows O_2_ rate measured by the electrode upon exposure of a lichen disk (4 mm diameter) to sub-saturating light intensity, 70 μmol m^−2^ s^−1^. A very small amount of O_2_ is detected directly after illumination starts, after which O_2_ rate gradually decreases over the next ~ 30 s followed by a linear increase until light is turned off. In the subsequent dark period, initially fast O_2_ consumption is followed by a more gradual decrease as the electrode continues to consume O_2_ in the dark. This results in a lower starting current for the second illumination trace and every subsequent trace (Illum 2 to 5, Fig. 2a). Each trace shows a similar pattern, where O_2_ rise becomes steeper for every subsequent illumination and so does the initial slope for O_2_ decrease (clearly visible comparing traces Illum 4 and 5 versus 1 and 2).

Figure 2b shows the same experiment for a dilute sample of the green alga *Chlorella vulgaris*. O_2_ response upon exposure to light is very different from the lichen response: a steep increase in O_2_ is observed directly upon illumination, followed by a more gradual increase up to a peak value of ~ 5000 nA. There is no lag phase and the O_2_ response pattern is repeated identically for subsequent illumination traces, apart from an offset in starting levels as a result of O_2_ consumption by the electrode during intermediate dark times. When normalized to Chl content, the O_2_ evolution activity is ~ 500 μmol O_2_ (mg Chl)^−1^ h^−1^, typical of Chlorella algae (Vinyard et al. 2013a, b).

**Fig. 3:**
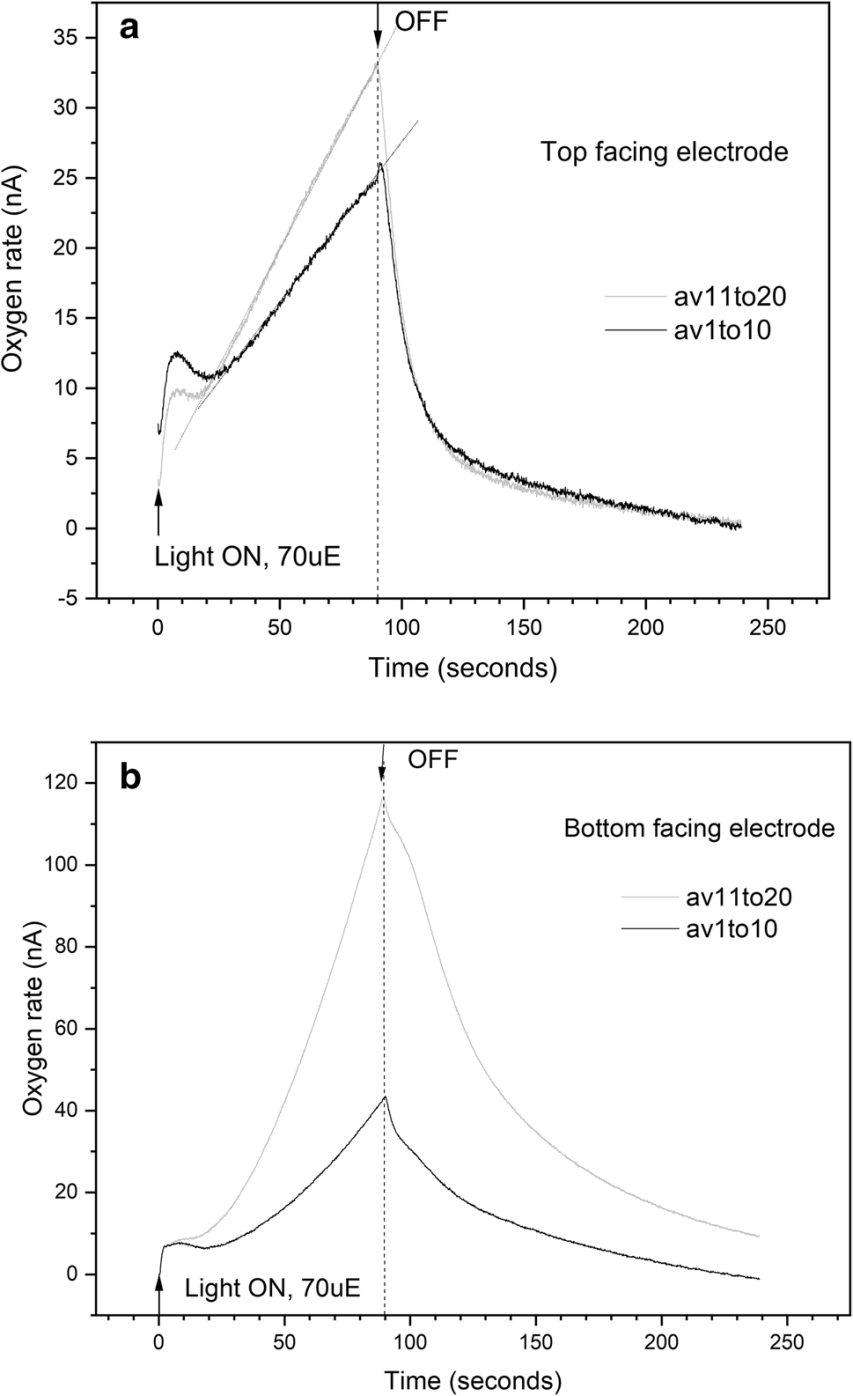
Time-resolved oxygen measured using the rate electrode (custom Clark electrode), from intact lichen disk (4 mm). Each continuous illumination period (light intensity 70 μmol m^−2^ s^−1^) lasted 90 s, separated by 10-min dark time between each illumination. Traces show mean of illumination periods 1 to 10 and 11 to 20. **a** Top of lichen disk facing electrode. **b** bottom of lichen disk facing electrode

Figure 3 shows time-resolved oxygen measured by the rate electrode for a lichen sample exposed to low light intensity (70 μmol m^−2^ s^−1^), comparing different orientations of the lichen disk towards the electrode. We compare results for upper surface facing the electrode, where the algal layer in the lichen thallus is situated, versus the bottom surface facing the electrode, which is exclusively fungal material (Honegger 1991). Traces shown here are averaged over 10 successive illuminations. Two transient features are observed within 30 s of light exposure, for both bottom and top facing the electrode. This is followed by a linear increase in O_2_ evolution that increases upon subsequent sets of illuminations by a factor of about 1.5 (top to electrode, Fig. 3a) and 3 (bottom to electrode, Fig. 3b), comparing mean of illuminations 1–10 versus 11–20. When light is turned off, the O_2_ flux decreases much less steeply when the bottom is facing the electrode compared to the rapid decrease when the top is facing the electrode, indicating a slower release of O_2_ from the thallus bottom. The average O_2_ current detected is 2–3-fold higher for the bottom facing electrode sample, which we attribute to biological variability in the field samples. Similar variability has been observed between samples with top facing electrode (SI.3). While absolute values vary between biological samples, repeated experiments with samples from different lichen thalli show a consistent 1.5- to 3-fold increase in the slope of linear O_2_ evolution, comparing mean of 10 illuminations, 11–20 versus 1–10 (data available in Supporting Information, Table SI.3.1).

**Fig. 4:**
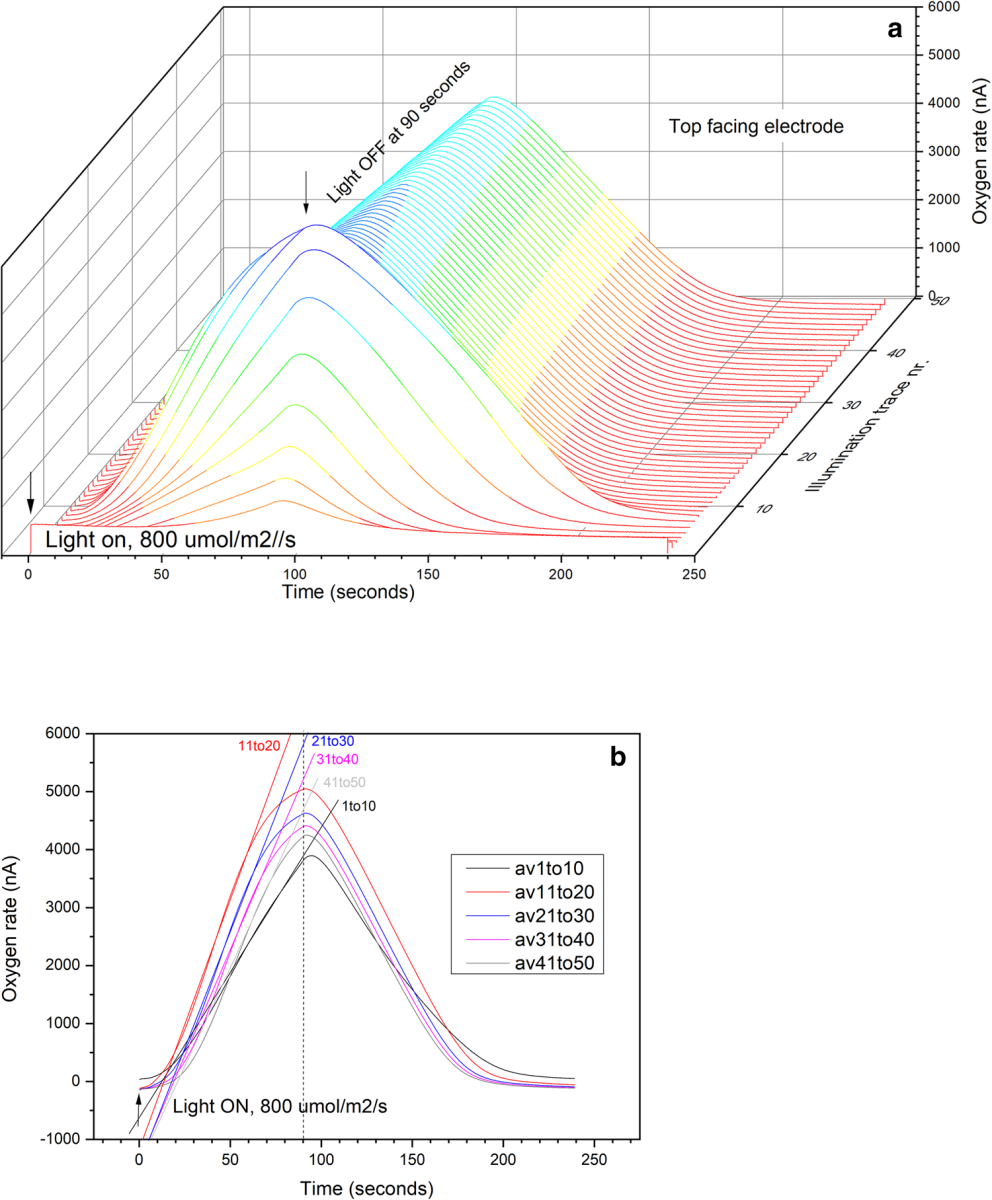
Time-resolved oxygen yield measured by custom rate electrode, from intact lichen disk (4 mm), light intensity 800 μmol m^−2^ s^−1^. **a** Traces show O_2_ yield for 50 successive illuminations, 90 s each. Color scale indicates changes in O_2_ yield (nA), corresponding to values indicated on vertical axis. **b** Traces show averages for sets of 10 traces, for the same experiment

A higher light intensity of 800 μmol m^−2^ s^−1^ was applied to test the O_2_ response at light intensity that normally saturates photosynthesis in free-living algae. The O_2_ yield for 50 successive illumination traces is presented in Fig. 4a, showing a steep increase in O_2_ amplitude over the first 8 traces (by a factor of 5.6, peak 1089 to 6054 nA), after which the amplitudes gradually decrease, to a peak value of 4184 nA at the 50th trace. Averages over sets of 10 illumination traces in Fig. 4b highlight three additional features: the slopes of O_2_ rate both during illumination and subsequently in the dark become steeper between the first and second sets of illuminations (1–10 and 11–20), while both slopes gradually decrease on subsequent traces (21–30, 31–40, 41–50). Additionally, the O_2_ production rate saturates before each trace completes, i.e., is no longer linear, indicating that a lower rate is reached. At this higher light intensity, a transition period is observed after light is turned off, where O_2_ rates continue to increase gradually until reaching a peak, then fall as O_2_ consumption becomes dominant. The timing of this point shifts closer to the point where light is turned off, comparing illuminations 1–10 versus subsequent sets of illuminations.

### Flash oxygen oscillations and absolute O_2_ yield in freed photobiont cells

**Fig. 5:**
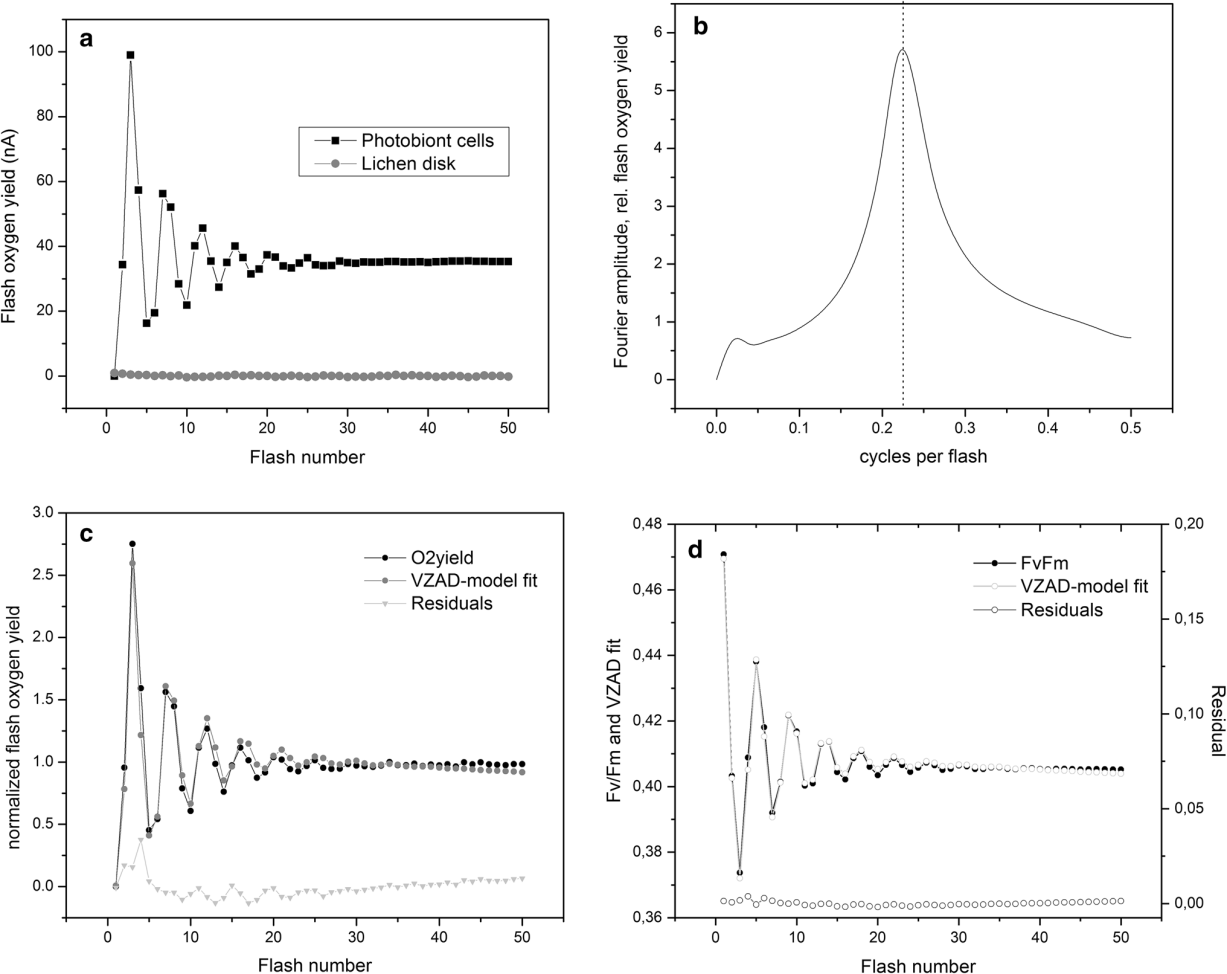
Flash O_2_ yields and electron transport from water oxidation measured by Chl. variable fluorescence (Fv/Fm), produced by single-turnover flashes (STF, 50 µs duration). **a** Flash O_2_ yield measured by custom rate electrode, from algal cells removed from lichen disk compared to yield from an intact lichen disk, using STF at a frequency of 0.5 Hz. **b** Fourier transformation of O_2_ oscillations from freed photobiont cells in (**a**), gives peak amplitude at 0.22 flash^−1^. **c** Least-squares fit of the experimental O_2_ flash yield in (**a**) to a standard WOC cycle model using the VZAD algorithm. Model-derived WOC cycle parameters available in SI.3. **d** Chlorophyll variable fluorescence (Fv/Fm) from a train of STFs applied to an intact, fully hydrated lichen disk, including least-squares fit to the VZAD model, showing normal period-4 oscillations from PSII water oxidation. Lower traces shows residuals between experiment and model

To gain further insight into the O_2_ production in lichen, we measured O_2_ yield in response to a train of single-turnover flashes (STF) from a lichen disk as well as from algal cells isolated from the same lichen disk. The lichen disk produced no detectable O_2_ over 50 flashes and no visible period-4 oscillations. (Fig. 5a). By contrast, isolated algal cells separated from the same lichen disk, produced strong period-4 oscillations in O_2_ yield in response to the sequence of STFs. Oscillations damped over 24–28 flashes to a high steady-state current of 36 nA or 40% oxygen yield relative to the peak amplitude (Fig. 5a). The undetectable flash O_2_ yield for intact lichen is consistent with continuous illumination measurements shown previously (Fig. 2a, b), accounting for the STF light on/off duty cycle (50 × 50 μs over 100 s duration, i.e., 5 × 10^−5^ times integrated light intensity compared to that for 90-s continuous illumination). Simulations of the decay of the oscillations using VZAD, a standard WOC cycle model (Vinyard et al. 2013a, b) confirm that the four-flash catalytic cycle of water oxidation is normal, typical of free-living algae. For reference, the WOC cycle inefficiency parameters for the VZAD fit are given in Supporting Material (Table 1 in SI.3). This confirms that algal cells in the lichen symbiosis are healthy and producing O_2_ at normal rates.

### The intrinsic electron transport rate from water oxidation measured by chlorophyll variable fluorescence

To directly verify that algal O_2_ production (water oxidation) actually occurs in intact lichens even though so little O_2_ is released at low light intensities, we measured period-4 oscillations of Chlorophyll variable fluorescence (Fv/Fm) in response to a train of STFs. To illustrate this, we show Fv/Fm response to 50 STFs from a lichen disk (7 mm diameter), in fully hydrated conditions (Fig. 5d). In addition to the steady-state amplitude of Fv/Fm ~ 0.41, typical of PSII in healthy light-adapted algal cells, we observe transient period-4 oscillations starting from dark-adapted samples, having typical amplitude of 1.1x the steady-state value (Vinyard et al. 2018).

## Discussion

### The role of photosynthetically produced O_2_ and sugars boosting lichen symbiosis

The observed increase in O_2_ consumption rates after prior illumination can be explained by the greater availability of sugars produced photosynthetically during illumination, being used for respiration. This observation is consistent with the literature showing that lichenized algae excrete sugars (ribitol, in alga *Trebouxia*) to the fungus to serve as electron donor for fungal respiration (Palmqvist 2000; Eisenreich et al. 2011). Dark respiration rates depend on availability of both O_2_ and reductant (NAD(P)H) at the locations where dark respiration takes place (mitochondria). The observation of higher linear respiration rates by the fungus after each successive illumination and at higher light intensities indicates that the delivery of reductant to the terminal respiratory enzyme is increased, causing the observed increase in electron flux to O_2_. Under anaerobic conditions (sealed container), the only source of O_2_ is that produced photosynthetically by the alga. This implies that increased fungal respiration rates following prior illumination, as observed in our experiments, confirm that both photosynthetic O_2_ (that we measure) and sugars (that are required to deliver reductant for respiration) are delivered by the alga to the fungus. Sources of NAD(P)H for respiration include catabolism of environmental carbohydrates and photosynthetic carbohydrates formed in the Calvin cycle (C3 to C6 sugars). The only source of exchangeable reductant that changes with illumination is photosynthetic carbohydrates produced by the alga.

Both O_2_ and/or sugars produced by the algae are used by the fungus, as this is the only source available to support an increase in respiration (ambient conditions being kept the same). Since no evaporation occurs (the lichen is immersed in water), only diffusional transport drives the flow of algal products (sugar and O_2_) through the lichen thallus and to the fungus. As this transport is much slower for sugar than for O_2_, the mostly likely source of increased fungal respiration at the time-scale of these experiments is O_2_. We conclude that the algal–fungal symbiosis encompasses both fungal consumption of algal sugars and the photosynthetically produced O_2_. This is the first report that we are aware of showing O_2_ exchange between lichen symbionts and its metabolic role in boosting respiration rates.

Internal O_2_ consumption by the lichen is confirmed by our complementary oximetry and fluorometry (Fv/Fm) experiments (Fig. 5). Although intact lichens release very low levels of O_2_, they exhibit normal period-4 oscillations in Fv/Fm, indicative of a normal WOC cycle (Vinyard et al. 2013a, b). The freed photobiont cells removed from the lichen exhibit normal O_2_ yield and period-4 oscillations in O_2_, typical of those found in many oxygenic phototrophs studied thus far, including *Flavoparmelia caperata* (Vinyard et al. 2018). Period-4 oscillations of Fv/Fm arise exclusively from water oxidation and demonstrate unequivocally that PSIIs in algal cells of hydrated lichens are fully active in O_2_ production (Ananyev and Dismukes 2005). Accordingly, the low yield of extracellular O_2_ from intact lichens at low light intensity is not due to an inactive PSII, but rather the algal O_2_ is consumed inside the lichen where it is directly available for reaction with the terminal respiratory enzymes during dark fungal respiration and potentially during algal photorespiration with RuBisCO.

Our results show that respiration rates not only substantially increase following illumination, but also are constant over time (linear) at external O_2_ concentrations above ~ 25 μ. The linearity can be explained by the available O_2_ concentration being above the reversible O_2_ binding affinity to the terminal respiratory enzymes of the fungus (Joseph-Horne et al. 2001; Aydin et al. 2017). By contrast, the gross photosynthetic O_2_ production rate does not vary substantially with extracellular O_2_ content (aerobic versus anaerobic). This is expected, since the WOC cycle is known to be irreversible and does not slow upon O_2_ partial pressures changing between zero and 20 bars (Kolling et al. 2009).

### Photosynthetic activity in wet lichens confirmed by period-4 oscillations in Fv/Fm and O_2_ production

Our experiments were conducted under water-saturated conditions, with lichen samples fully hydrated or immersed in water in the sample cuvette. Previous studies have shown depression of photosynthetic activity in some, but not all lichen species (Lange et al. 1993, 1996, 2001, 2006, 2007; Lange and Green 1996). Full photosynthetic activity of our lichen samples is confirmed by our measurements of period-4 oscillations in Fv/Fm and oximetry experiments, for fully hydrated lichen samples. Active period-4 oscillations indicate an active Water Oxidation Complex (WOC) capable of producing O_2_ (Fig. 5 and Figure SI.1). Full activity of the WOC is further confirmed by good quality fits to the WOC cycle model (VZAD) (Figure SI.4). Previous experiments investigating photosynthetic activity in relation to thallus water content were based on measurements of CO_2_ exchange, relying on gas exchange between the sample and its surroundings. Decrease in CO_2_ exchange under water-saturated conditions may be explained by delayed gas transport from the sample. By contrast, our measurements of Fv/Fm enable direct measurement of photosynthetic activity independent of gas exchange rates and confirm full photosynthetic activity of the lichen samples under supersaturated conditions.

### Transient electron transport kinetics in lichen photosynthesis

**Fig. 6:**
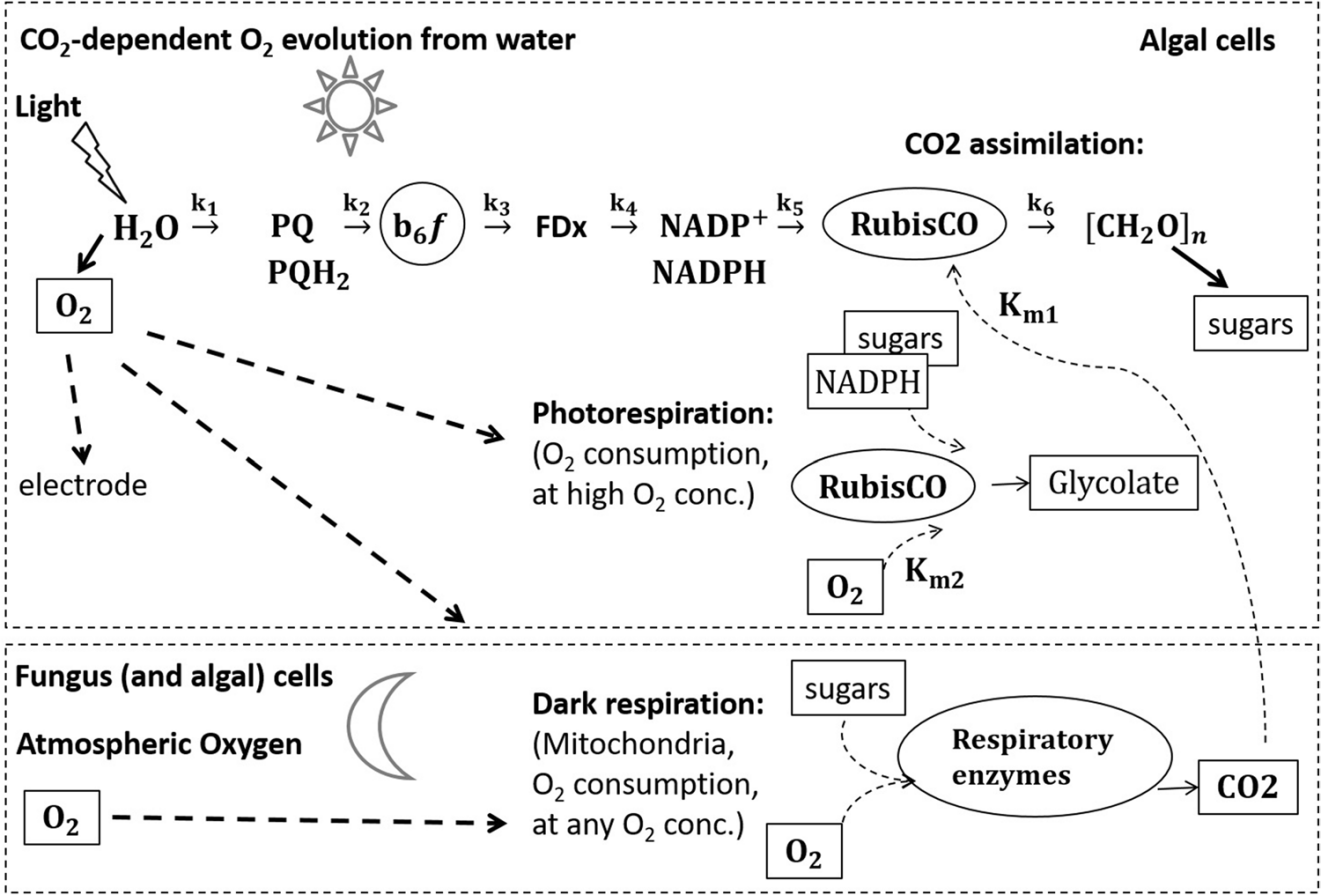
Sequence of electron transport in relation to oxygen formation in the WOC, CO_2_ assimilation and relation with O_2_ consumption by dark respiration, photorespiration, and the electrode. *PQ* Plastoquinol, *b*_*6*_*f* cytochrome, *FDx* ferrodoxin. k_1_ to k_6_ are fluxes, K_m1_ and K_m2_ are the Michaelis constants for CO_2_, respectively, O_2_ binding at RuBisCO

The transient O_2_ features observed by the rate electrode, at 10-fold faster time resolution (0.1–0.3 s), are all accounted for by linear electron flow from water to successive downstream electron acceptors (as illustrated in Fig. 6). An initial minimum in O_2_ rate reached in 1–2 s (Figs. 2a, 3) corresponds to the time it takes to fill the plastoquinol (PQ) pool with electrons from PSII water oxidation (k_1_ in Fig. 6). The following local maximum at ~ 10 s corresponds to the time for partially emptying electrons from the PQ pool at this light-limited rate by the slower PSI flux into the pool of terminal electron acceptors of PSI (NADP^+^ and Ferredoxin pools, k_2_ to k_4_ in Fig. 6). A second local minimum occurs when this pool is filled. The O_2_ transients are followed by a linear positive slope which corresponds to transfer of the electrons to the terminal acceptor pool of CO_2_ via RuBisCO (CO_2_-dependent O_2_ evolution limited by the rate of RuBisCO turnover, Fig. 2a). The dependence of this slope on CO_2_ concentration has been demonstrated previously in free-living algae, where it is followed at much longer times by decrease in the CO_2_-dependent O_2_ evolution rate as the co-factors needed to fix CO_2_ (CO_2_, NADPH and ATP) are depleted (k_5_ and k_6_ in Fig. 6) (Ananyev et al. 2016a, b). The latter decrease is not evident in the 4-min illumination period used for our lichen samples. All of these transients were previously identified by Chl variable fluorescence spectroscopy of free-living algae and are common to all photosynthetic electron transport chains (Ananyev et al. 2016a, b).

### Oxygen production in response to CO_2_ availability

The observed increase in slope of the linear CO_2_-dependent phase of O_2_ evolution (Figs. 3, 4) can be explained, in principle, by either a O_2_ source (increase in O_2_ production) or sink (decrease in O_2_ consumption) mechanism. An increase in photosynthetic O_2_ production rate (electron transport: H_2_O →→ NADP^+^ → CO_2_) can arise if the terminal electron acceptor concentration increases, owing to the greater amount of CO_2_ coming into the alga from fungal respiration. In turn, the increased fungal respiration originates from the increased Calvin-cycle sugars excreted by the alga to the fungus during successive pre-illumination cycles. Greater availability of photosynthetic O_2_ within the lichen is not expected to increase fungal respiration alone (without co-release of sugar), for O_2_ concentrations above saturation.

Alternatively, lower O_2_ consumption (respiration or binding) by the fungus can increase the slope of the linear CO_2_-dependent phase of photosynthetic O_2_ evolution with successive illuminations. However, O_2_ consumption rates in the dark increase upon subsequent illuminations (Figs. 1, 3). The increase in O_2_ consumption rates continues until saturation is reached, after which both O_2_ production and consumption rates gradually become less steep (Fig. 4). Furthermore, the sink mechanism predicts a non-linear rising slope of light-induced O_2_ evolution detected at the electrode as the O_2_ consuming sites get saturated, which is not observed (the slopes are linear and reach saturation within the 90-s illumination interval). Only after multiple illumination cycles, beyond 20–30 illumination cycles at high light intensity, does the net photosynthetic O_2_ evolution rate decreases which we attribute to consumption of fungal-respired CO_2_ (Fig. 4a, b). This light saturation occurs at much higher light intensity compared to free-living algae, which typically saturate at much lower light fluxes in all eukaryotic algal taxa, for example, 12 μmol m^−2^ s^−1^ for red alga (Terada et al. 2016); 40–240 μmol m^−2^ s^−1^ for brown algae (Borlongan et al. 2018); and < 100 μmol m^−2^ s^−1^ for green algae (Falkowski and LaRoche 1991).

The increase in algal water oxidation rate that is linked to higher internal CO_2_ availability produced by fungal respiration demonstrates a new metabolic linkage between the symbionts in lichens that further expands the scope of what constitutes symbiosis in general. The lichen symbionts exchange not only water and sugar, as known before, but also CO_2_ and O_2_, as found herein.

### Carbon concentrating mechanism

The CO_2_ component of this symbiosis is a form of Carbon Concentration Mechanism (CCM) that is induced by internal CO_2_ production and delivery from the mycobiont to the photobiont in order to boost O_2_ production under low internal CO_2_ conditions. Its simultaneous O_2_ and CO_2_ exchange between symbionts distinguishes it from CCMs that exist in free-living cyanobacteria and algae, where internal CO_2_ stores are filled during illumination and subsequently released in the dark (Badger et al. 2005). These concentrating mechanisms are based on active dissolved inorganic carbon (DIC)-uptake processes which are energized by photosynthetic electron transport and may capture either CO_2_ or HCO_3_^−^ from the external environment. A CCM has been observed to operate in cyanobacterial lichens which is capable of considerable elevation of internal CO_2_ and is “similar to that reported for free-living cyanobacteria” (Badger et al. 1993). However, these measurements using CO_2_ gas exchange between the atmosphere and lichens do not reveal whether an inter-species CCM is operative and they attribute the observation exclusively to an internal mechanism within the photobiont. The authors observe a considerably smaller CCM in a green algal lichen (measured as a 10-fold smaller pool of CO_2_ released in darkness after illumination) and suggest that “it is probably less effective than that which operates in cyanobacterial lichens.” By contrast, our results using oximetry and fluorometry indicate that this interpretation—a single organismal source mechanism to account for the much smaller pool of CO_2_ released in darkness after illumination in green algal lichens—is insufficient and actually arises from the much greater capacity of the green algal photobiont to consume fungal-generated CO_2_ by an inter-species exchange mechanism.

### Photorespiration

The delay in occurrence of peak O_2_ after light is turned off that occurs especially at high light intensity (Fig. 4) suggests that the larger amount of photosynthetic O_2_ produced at the higher light intensity results in increased competition between O_2_ and CO_2_ within the alga for reduction by RuBisCO. We attribute this to the well-known photorespiration reaction observable in free-living phototrophs (Fig. 6), which favors the oxygenase reaction over the carboxylation reaction at increasing light intensity owing to the greater amount of O_2_ available (Somerville 2001).

## Conclusions

In this work, we investigated oxygen production and respiration in lichen *Flavoparmelia caperata*, aiming at a deeper understanding of the role of oxygen produced by the photobiont and CO_2_ produced by the fungus in the symbiotic relationship. We discovered the first evidence that photosynthetic O_2_ and respiratory CO_2_ mutually power the lichen symbiosis, together with the previously recognized exchange of Calvin-cycle sugars and water. The higher rates of algal photosynthesis that occur upon repeated light exposure, both O_2_ production and CO_2_ fixation, stimulate correspondingly faster rates of fungal respiration in darkness. The respiratory consumption of photosynthetic O_2_ and sugars by the fungus in turn boosts the algal CO_2_-dependent O_2_ evolution rate significantly, such that minimal light saturation of photosynthetic flux from water (O_2_ evolution) to CO_2_ occurs at light intensities that would completely light-saturate O_2_ evolution in free-living algal cells. We conclude that the algal–fungal symbiosis of lichens is mutually beneficial to the metabolism of both organisms at the fundamental level of electron transport in both photosynthesis and dark respiration, including electron transport for both metabolisms (H_2_O and CO_2_ for photosynthesis, sugars and O_2_ for respiration). The algal and fungal energy metabolisms are mutually linked, creating a two-way turbo-charged symbiosis.

## Electronic supplementary material

Below is the link to the electronic supplementary material.

Supplementary material 1 (DOCX 2146 kb)
