# Supplementary material for: Symbiosis extended: exchange of photosynthetic O2 and fungal-respired CO2 mutually power metabolism of lichen symbionts
Source: Photosynth Res. 2019 Dec 31;143(3):287–99. doi: 10.1007/s11120-019-00702-0 (PMC7052035; doi:10.1007/s11120-019-00702-0)
Supplement: Supplementary file 1 — Supplementary material 1 (DOCX 2146 kb) [file 11120_2019_702_MOESM1_ESM.docx]

**SUPPORTING INFORMATION**

**Article title:**

**Symbiosis extended: exchange of photosynthetic O_2_ and fungal-respired CO_2_ mutually power metabolism of lichen symbionts**

**Photosynthesis Research**

**Corresponding author: Marie-claire ten Veldhuis, Delft University of Technology (j.a.e.tenveldhuis@tudelft.nl)**

**Co-authors: Gennady Ananyev, Gerard Charles Dismukes**

**SI.1 Photosynthetic activity in lichen *Flavoparmelia caperata* under water-saturated conditions**

Figure SI.1.1 shows that Fv/Fm is constant when saturated with water condition (216% WC, ratio wet weight/dry weight) and shows no depression, but remains constant as lichens gradually dehydrate from saturation down to a critical water content (~85% WC), where Fv/Fm starts to decrease until it becomes negligible when lichens are dry (~10% WC).


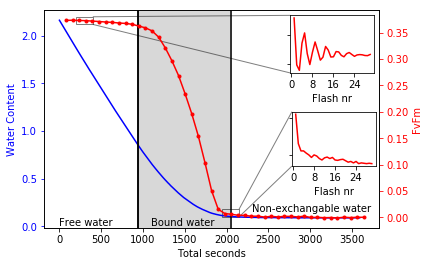

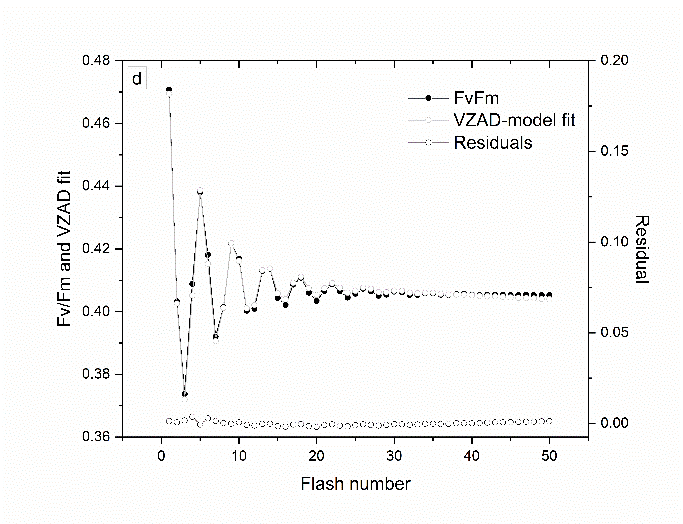


Figure SI.1.1 Fv/Fm in lichen Flavoparmelia caperata samples. a. Fv/Fm (in red) and water content (in blue) measured over time for a wet sample gradually evaporation as it is exposed to atmospheric RH 0.34%. Insets show period-4 oscillations in Fv/Fm for the sample in wet (strong period-4 oscillations) and in dry state (period-4 oscillations lost). Active period-4 oscillations indicate active water splitting in the Water Oxidizing Complex (WOC).

b. Fv/Fm period-4 oscillations in a sample immersed in water (50 µl water added to a 7 mm diameter lichen sample equivalent to ~1000% WC (wet weight relative to dry weight)). Period-4 oscillations show a good fit to the VZAD model with very small residuals. More details on VZAD are provided in SI4.

**SI.2 Experimental results O_2_ production and respiration from lichen, under aerobic and anaerobic conditions**

**Table SI.2.1** Lichen O2 production and respiration rates (µM/hour).

| **Initial conditions** | **Aerobic** (μM/hour) | | | | **Anaerobic** (μM/hour) | | | |
| --- | --- | --- | --- | --- | --- | --- | --- | --- |
| **O_2_ response trace** | **D1** | **L1** | **D3** | **L1-D3** | | **L2** | **D6** | **L2-D6** |
| Experiment nr. (1) | -215 | 275 | -340 | 615 | | 375 | -240 | 615 |
| nr. (2) | -660 | 550 | -1090 | 1640 | | - | - | - |
| nr. (3) | -460 | 390 | -455 | 845 | | 518 | -295 | 813 |
| nr. (4) | -385 | 320 | -580 | 900 | | 392 | -650 | 1042 |


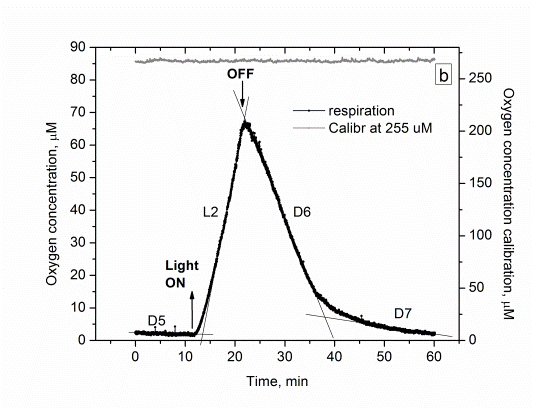

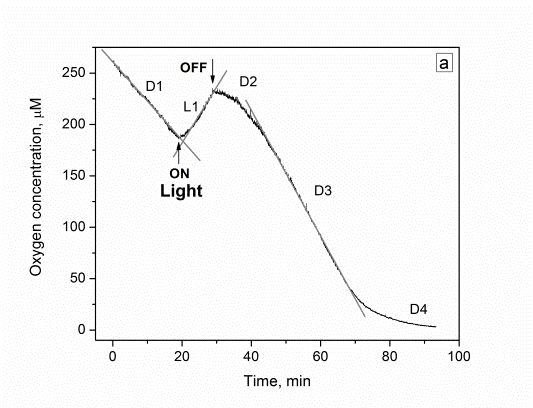

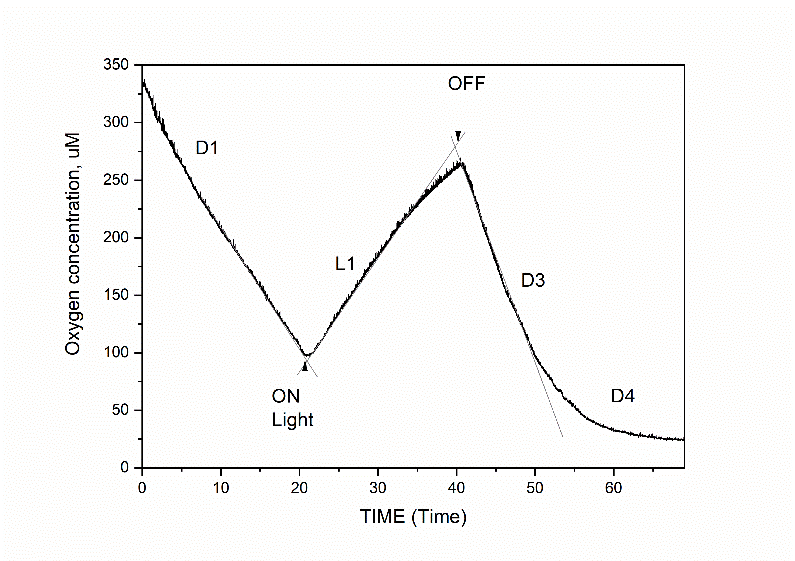


**Figure SI.2.1a** Evolution of extracellular O_2_ concentration in Clark cell chamber (1 ml) holding 13 lichen disks (4mm diameter each) and stirred vigorously. Figures corresponding to experiments nr 1 and 2. Figures in left column: Initial conditions aerobic, at O_2_ saturation in water (O_2_ ~ 255 μM) and following ~10 min pre-measurement dark period of the ambient exposed lichen. Figures in order corresponding to numbers in Table SI.1.1: first row, experiment nr. (1), 2^nd^ row experiment nr 2.


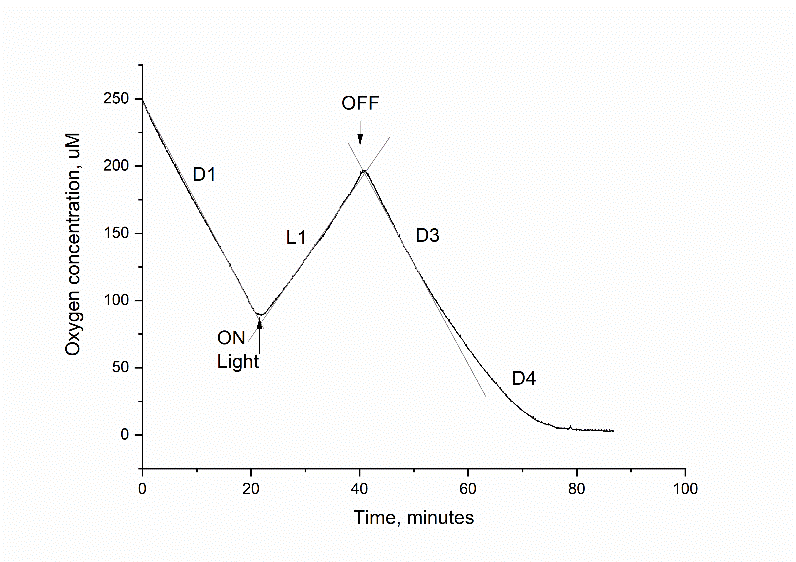

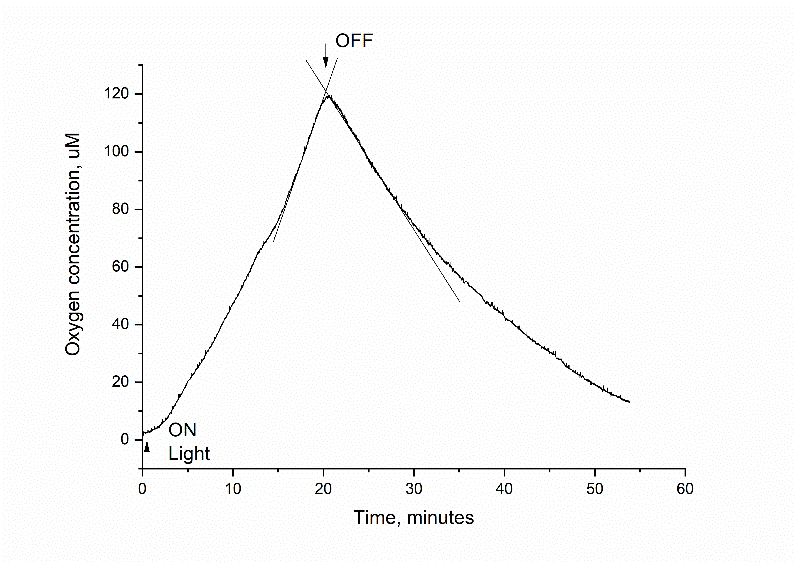

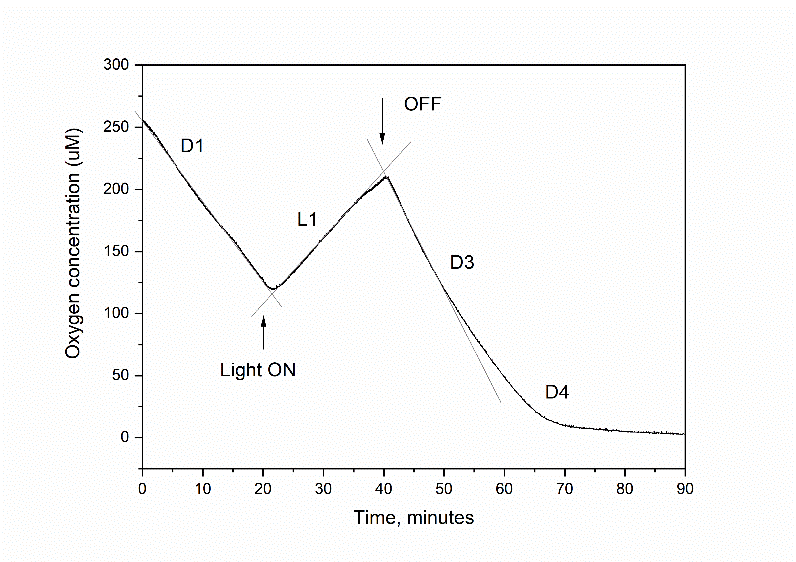

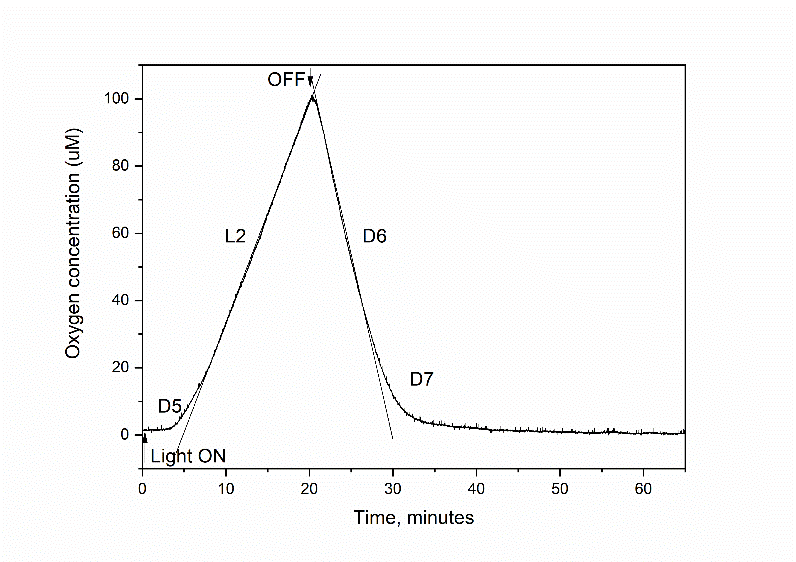


**Figure SI.2.1b** Evolution of extracellular O_2_ concentration in Clark cell chamber – experiments nr. 3 and 4. Figures in left column: Initial conditions aerobic, at O_2_ saturation in water (O_2_ ~ 255 μM) and following ~10 min pre-measurement dark period of the ambient exposed lichen.

Figures in order corresponding to numbers in Table SI.1.1: first row, experiment nr. (3), 2^nd^ row experiment nr 4.

**SI.3 Experimental results transient oxygen flux, for different samples and at different light intensities**

**Fig. SI.3.1a** Experiment nr 1, sample 0146 **Fig. SI.3.1b** Experiment nr 2, sample 0147


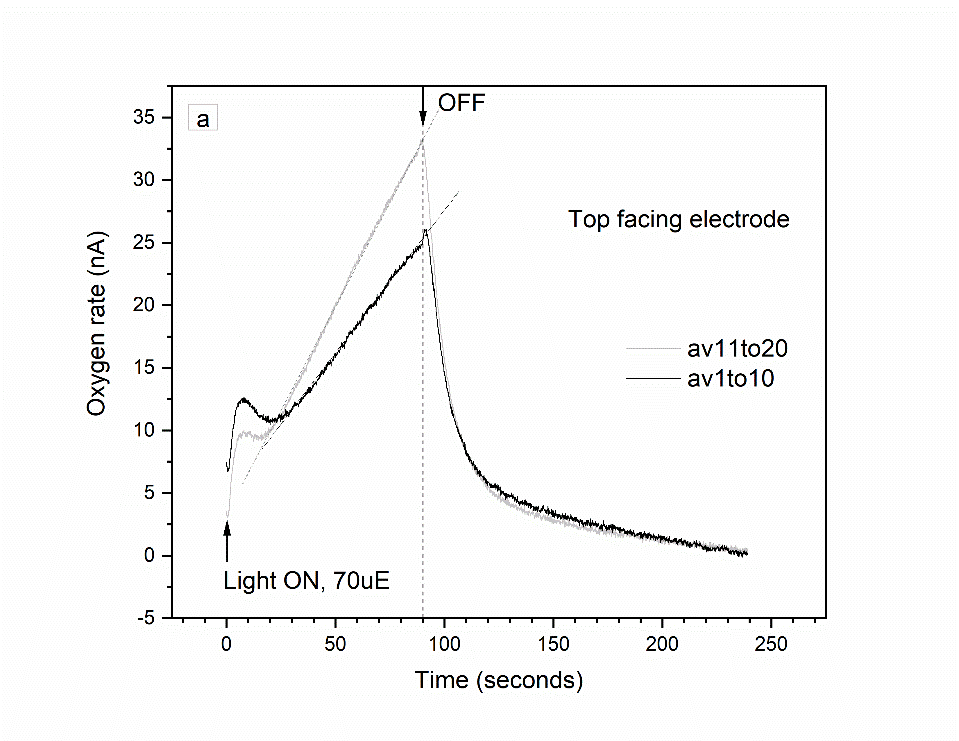

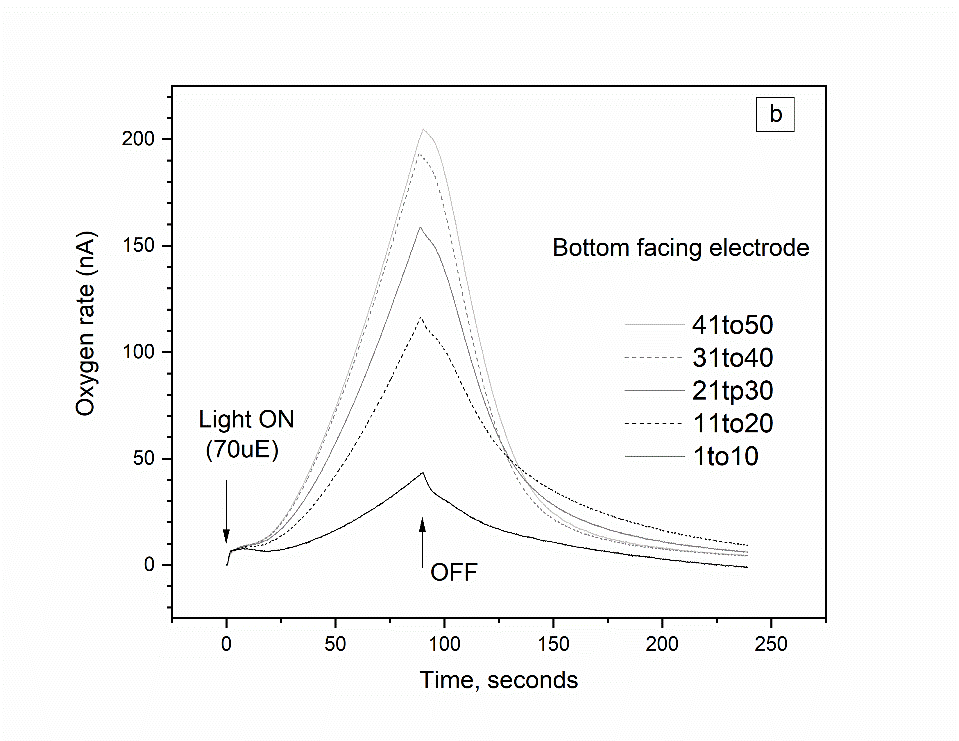


**Fig. SI.3.2a** Experiment nr 3, sample 004 **Fig. SI.3.2b** Experiment nr 4, sample 201


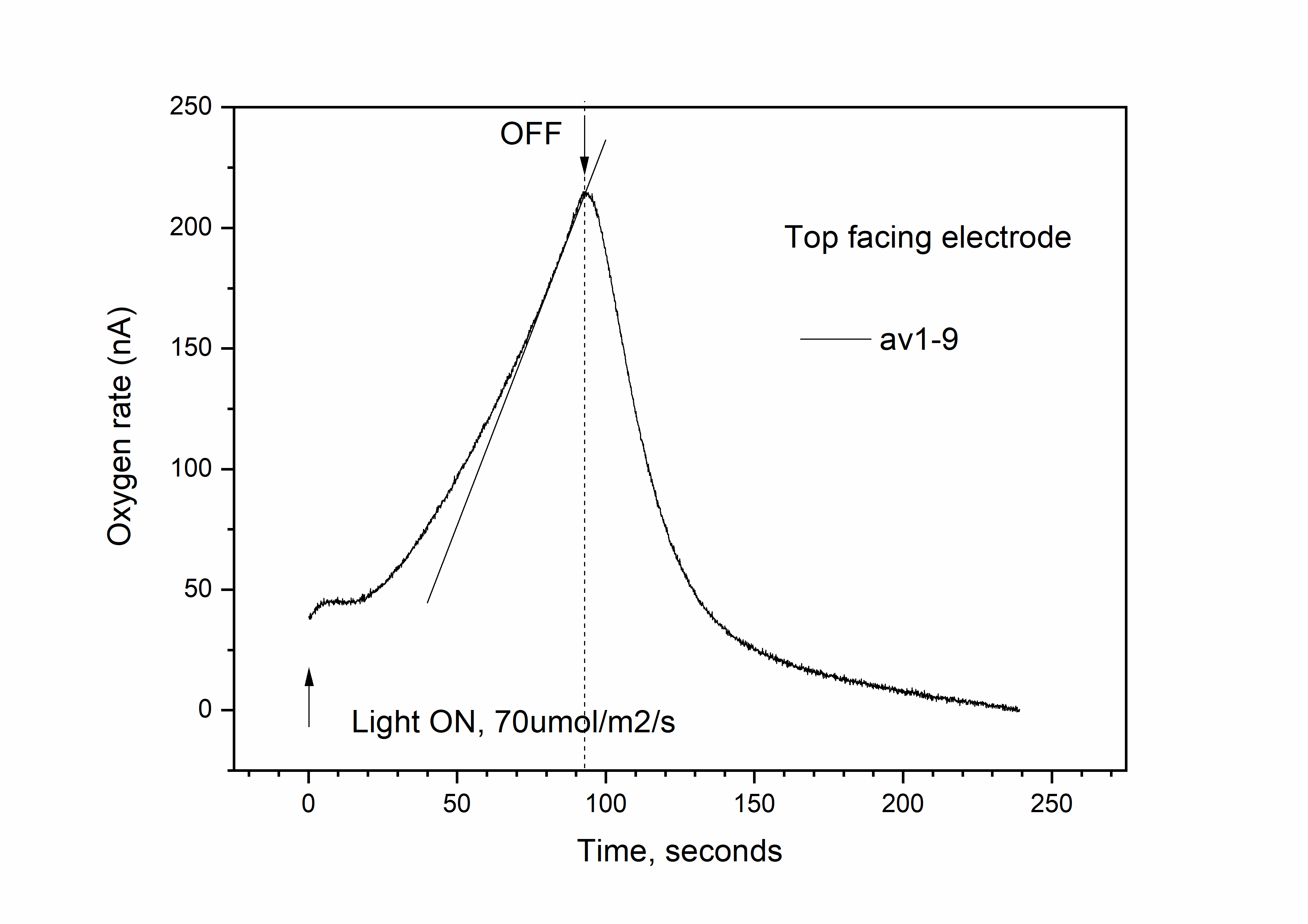

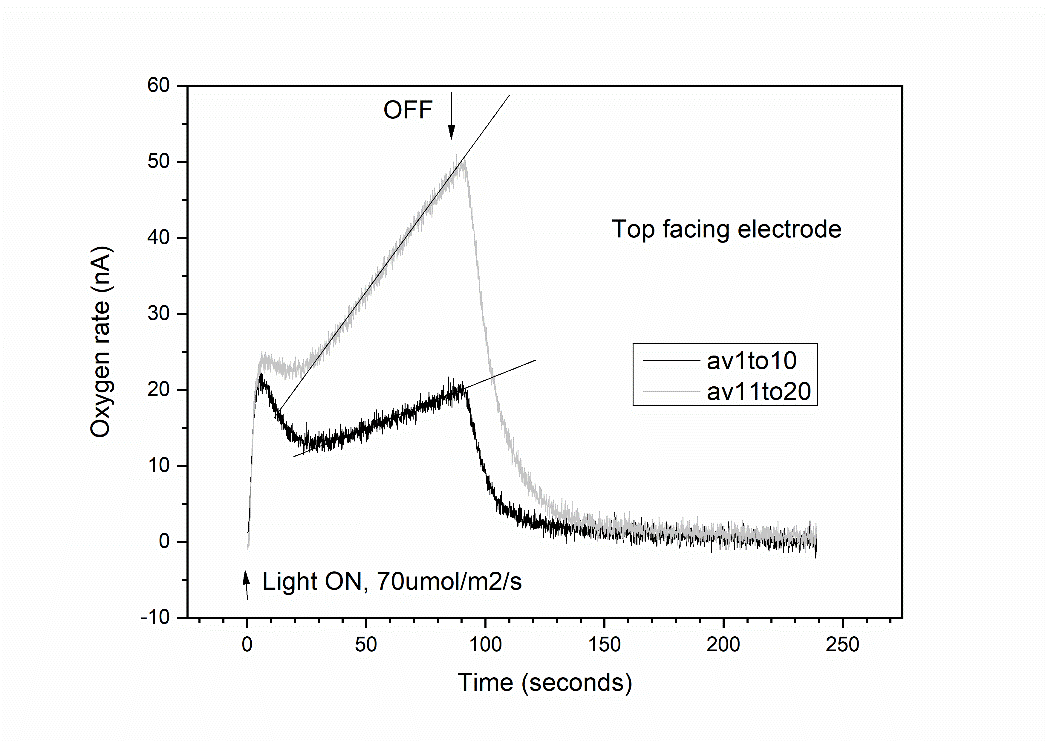

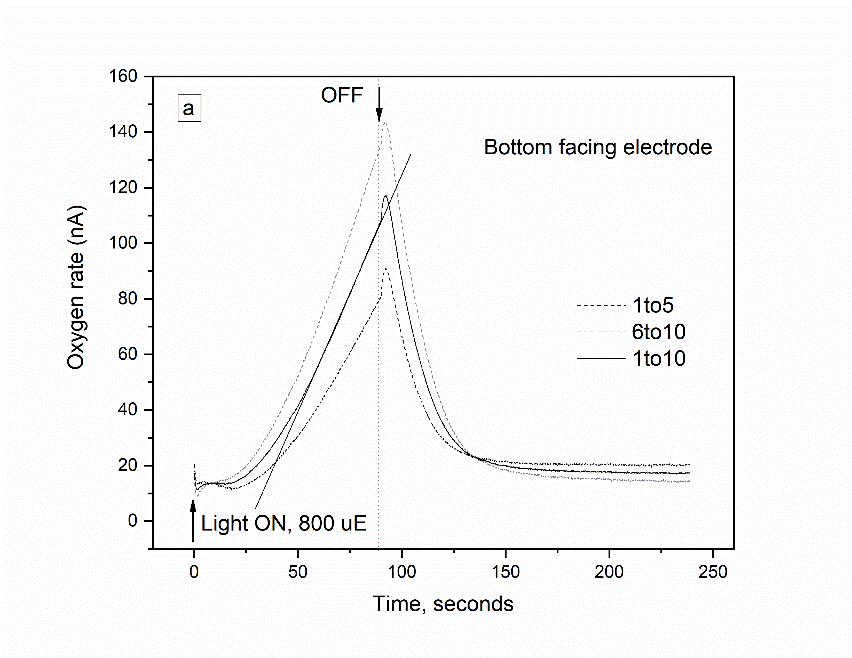


**Fig. SI.3.2a** Experiment nr 5, sample 0146a **Fig. SI.3.2b** Experiment nr 6, sample 004a


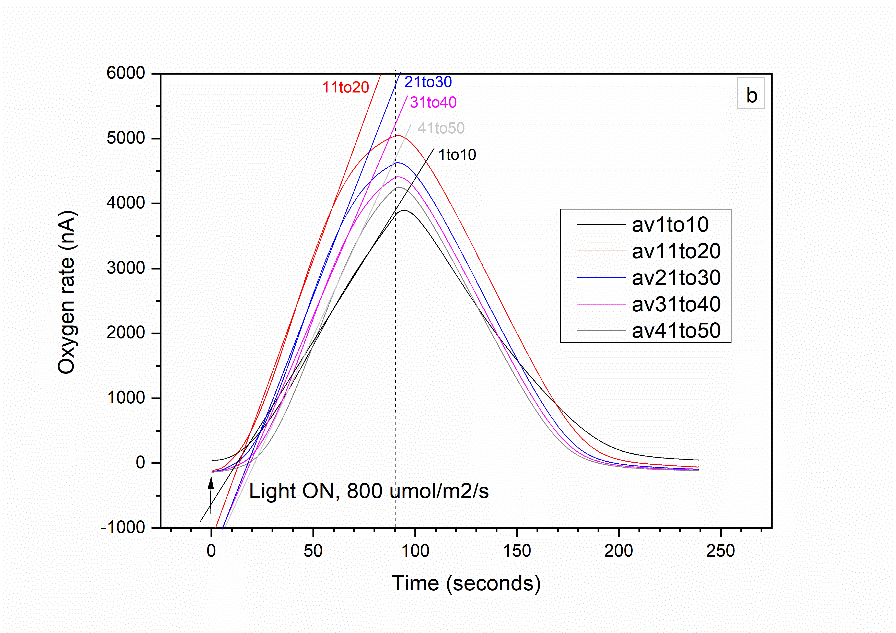


**Fig. SI.3.4** Experiment 7, sample 201


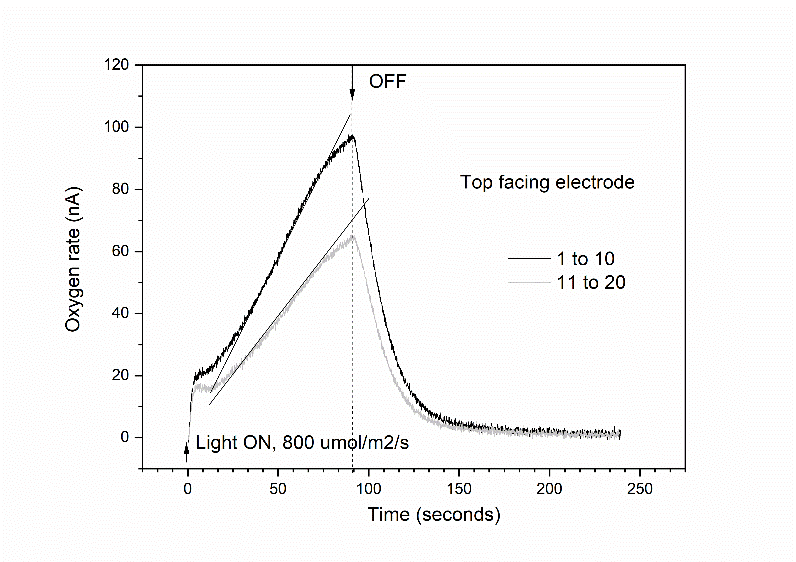


**Table SI.3.1** Transient O_2_ fluxes in lichen at 70 µmol.m-2.s-1 and 800 µmol.m-2.s-1 light intensities. Fluxes are measured as current (nA) generated by O_2_ consumption at the electrode. T2El, B2El: top resp. bottom of lichen sample facing electrode.

| **Experiment and sample number** | **Light**  **Intensity, sample position** | **Mean of Traces** | **Slope linear O2 prod (nA/s)** | **Relative slope increase after illuminations** | **Slope linear O2 resp (nA/s)** | **Relative slope increase after illuminations** |
| --- | --- | --- | --- | --- | --- | --- |
| (1)  s0146 | 70uE,  T2El. | 1-10 | 0.25 | REF | -1.3 | REF |
|  |  | 11-20 | 0.37 | 1.5x | -1.7 | 1.3x |
| (2)  s0147 | 70uE  B2El. | 1-10 | 0.76 | REF | -0.4 | REF |
|  |  | 11-20 | 2.1 | 2.8x | -1.5 | 3.8x |
|  |  | 21-30 | 2.7 | 3.6x | -2.7 | 6.8x |
|  |  | 31-40 | 3.0 | 3.9x | -3.7 | 9.3x |
|  |  | 41-50 | 3.5 | 4.6x | -4.0 | 10.0x |
| (3)  s004 | 70uE,  T2El | 1-4 | 1.4 | REF | -2.9 | REF |
|  |  | 5-9 | 3.9 | 2.8x | -7.6 | 2.6x |
| (4)  s201 | 70uE,  T2El | 1-10 | 0.14 | REF | -1.5 | REF |
|  |  | 11-20 | 0.36 | 2.6x | -2.2 | 1.5x |
| (5)  s0146a | 800 uE  B2El. | 1-5 | 1.4 | REF | -3.4 | REF |
|  |  | 6-10 | 2.1 | 1.6x | -5.6 | 1.6x |
| (6)  s004a | 800uE,  T2El | 1-10 | 40 | REF | -41 | REF |
|  |  | 11-20 | 73 | 1.8x | -50 | 1.2x |
|  |  | 21-30 | 70 | 1.7x | -46 | 1.1x |
|  |  | 31-40 | 63 | 1.6x | -44 | 1.2x |
|  |  | 41-50 | 56 | 1.4x | -43 | 1.0x |
| (7)  s201a | 800uE  T2El | 1-10 | 0.6 | REF | -3.5 | REF |
|  |  | 11-20 | 1.0 | 1.7x | -2.6 | 0.7 |

**SI.4 Flash O2 yield, Fourier transform and VZAD model fit**

**Table SI.4.1** Parameters VZAD model fit and Fourier transform for O2 period-4 oscillations of algal cells removed from lichen disk.

| **VZAD model parameters** | **Values model fit** |
| --- | --- |
| Alpha: | 0.147 |
| Beta: | 0.0076 |
| Delta: | 0 |
| Epsilon: | 0.015 |
| **Fourier Transform (FT) parameters** | **Values** |
| Theor FT Period: | 4.468 |
| Fit FT Period: | 4.462 |
| FT peak amplitude frequency | 0.22 flash^-1^ |
| Fit FT area: | 0.898 |
| S0Norm: relative population | 0.045 |
| S1Norm: relative population | 0.756 |
| S2Norm: relative population | 0.198 |
| S3Norm: relative population | 0 |





**Figure SI.4.1** Normalised flash O_2_ yield (in black) from algal cells removed from lichen disk, measured by custom Clark electrode, using Single Turnover Flashes (STF) at a frequency of 0.5 Hz. Normalisation by mean value over last 10 STFs (36 nA). Period-four oscillations in O2 yield indicate the presence of active PSII in isolated algal cells. Least-squares fit of the experimental O_2_ flash yield (in grey) to a standard WOC cycle model using the VZAD algorithm (Vinyard, Zachary, Ananyev, & Dismukes, 2013). Model-derived WOC cycle parameters are reported in Table SI.1.





**Figure SI.4.2** Fourier transformation of O_2_ oscillations from freed photobiont cells in Figure SI.1. Parameters of Fourier transform are reported in Table SI.1.

**Reference**

Vinyard, D., Zachary, C., Ananyev, G., & Dismukes, G. (2013). Thermodynamically accurate modeling of the catalytic cycle of photosynthetic oxygen evolution: a mathematical solution to asymmetric Markov chains. *Biochimica et Biophysica Acta (BBA)-Bioenergetics, 1827*(7), 861-868.
